# Overexpression Of Hepatocyte Nuclear Factor-1beta Predicting Poor Prognosis Is Associated With Biliary Phenotype In Patients With Hepatocellular Carcinoma

**DOI:** 10.1038/srep13319

**Published:** 2015-08-27

**Authors:** Dan-Dan Yu, Ying-Ying Jing, Shi-Wei Guo, Fei Ye, Wen Lu, Quan Li, Yu-Long Dong, Lu Gao, Yu-Ting Yang, Yang Yang, Meng-Chao Wu, Li-Xin Wei

**Affiliations:** 1 Tumor Immunology and Gene Therapy Center, Eastern Hepatobiliary Surgery Hospital, The Second Military Medical University, Shanghai, China; 2 Department of Comprehensive Treatment, Eastern Hepatobiliary Surgery Hospital, The Second Military Medical University, Shanghai, China

## Abstract

Hepatocyte nuclear factor-1beta (HNF-1B) is involved in the hepatobiliary specification of hepatoblasts to cholangiocytes during liver development, and is strongly expressed throughout adult biliary epithelium. The aim of this study was to examine the expression of HNF-1B in different pathologic subtypes of primary liver cancer, including hepatocellular carcinoma (HCC) and cholangiocarcinoma (ICC), and the relationship between HNF-1B expression, clinicopathological features and prognosis. We retrospectively investigated 2 cohorts of patients, including 183 HCCs and 69 ICCs. The expression of HNF-1B was examined by immunohistochemistry. We found that HNF-1B expression was associated with pathological subtype of primary tumor, and HNF-1B expression in HCC tissue may be associated with the change of phenotype on recurrence. The HNF-1B expression was positively correlated with biliary/HPC (hepatic progenitor cell) markers expression. Further, multivariable analysis showed that HNF-1B expression was an independent prognostic factor for both overall survival and disease-free survival of HCC patients. However, no correlation between HNF-1B expression and survival was found in ICC patients. In summary, HCC with high HNF-1B expression displayed biliary phenotype and tended to show poorer prognosis. HNF-1B-positive malignant cells could be bipotential cells and give rise to both hepatocytic and cholangiocytic lineages during tumorigenesis.

The World Health Organization (WHO) classifies primary liver cancers into hepatocellular carcinoma (HCC), intrahepatic cholangiocarcinoma (ICC), and combined hepatocellular-cholangiocarcinoma (CHC)^1^. Hepatocellular carcinoma (HCC) is the fifth most common cancer in the world and the third leading cause of cancer-related deaths worldwide^2^. HCC represents the major histological subtype of primary liver cancers. ICC is the second most frequent type of liver cancer and its incidence has increased drastically over the past two decades^3^.

Cholangiocyte transcription factors such as hepatocyte nuclear factor-1beta (HNF-1B), also called variant HNF1 (vHNF1), is a homeodomain protein that plays an essential role in the liver-specific expression of many genes during differentiation and development^4^. In human, HNF-1B was first described to be associated with disease in 1997 that heterozygous germline mutations in HNF-1B cause maturity-onset diabetes of the young, subtype 5(MODY5)^5^. In adults, HNF-1B is strongly expressed in the biliary system and is also expressed in the periportal hepatocytes^6^. HNF-1B is also reported to regulate the expression of genes associated with stem/progenitor cells, including osteopontin (OPN), Cluster of differentiation 24 (CD24) and CD44, which indicates that HNF-1B may play an important role in stem cell regulation^7^,^8^. Recent studies have also shown that expression of HNF-1B is associated with higher risk of HCC. Analysis of the expression of HNF-1α and HNF-1B mRNA in HCC tissues showed that the ratio of HNF-1α/HNF-1B mRNA is higher in well-differentiated cases than in poorly-differentiated and undifferentiated cases^9^. Shim *et al.*^10^ reported that HNF-1B predicts recurrence and HCC-specific death after transplantation in patients with HCC. However, the potential mechanism is still not clear.

Studies reported that HCC with biliary differentiation, defined as having cytokeratin (CK) 19 positive cells, tended to show poorer surgical outcome^11^. It has recently been proposed that HCC with biliary differentiation should be categorized as HCC with stem/progenitor cell immunophenotype. Furthermore, many studies have provided the evidence that HCC with hepatic progenitor cell (HPC) markers expression had poor prognosis^12^,^13^,^14^. Therefore, we hypothesized that as a biliary marker, HNF-1B expression in primary liver cancer would be associated with HPC markers expression and predict a poorer outcome. So far, the study of HNF-1B and liver cancer is still limited. To the best of our knowledge, the association between HNF-1B expression and the histopathologic type of liver cancers, and the prognostic value of HPC/biliary markers for HCC have not been studied in detail.

Therefore, we performed a clinicopathological study on patients who had undergone hepatectomy for histologically proven HCC and ICC. We investigated HNF-1B expression in different pathologic subtypes of primary liver cancers, and the relationship between HNF-1B expression and HPC/biliary markers expression by means of immunohistochemistry.

## Results

### Clinical and pathological features

The clinical and pathological characteristics of HCC and ICC patients were summarized in Table 1. All the patients underwent curative liver resection of primary liver cancer, and did not receive any other treatments before surgery. Diagnosis of HCC or ICC was made under Hematoxylin & Eosin (HE) staining in all patients of the 2 cohorts. We found that 15 of the 183 patients in cohort 1 were diagnosed as HCC primarily at the first surgery while their recurrence changed to ICC in the subsequent surgeries. The recurrent time of these 15 patients was all within 2 years.

### The association of HNF-1B expression in primary tumor with different pathologic subtypes of recurrent tumor

**Figure 1:**
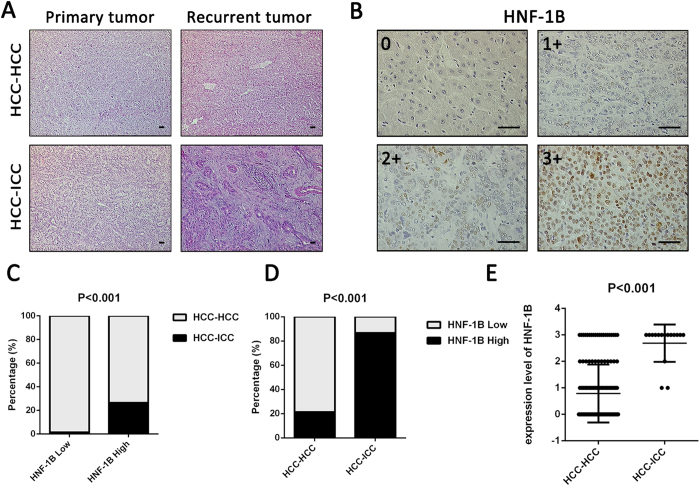
HNF-1B expression in primary tumor is associated with different pathologic types of recurrent tumor. (**A**) Representative images in primary tumor and recurrent tumor of HE staining. (HCC-HCC: the non-pathological transition group. HCC-ICC: the pathological transition group, primary tumor was HCC while recurrent tumor changed to ICC) (sacle bar = 100 μm). (**B**) Representative images in primary tumor of HNF-1B immunostaining in 0, 1+, 2+ and 3 + (sacle bar = 100 μm). (**C**) The percentage of pathological transition cases in HNF-1B low expression group and HNF-1B high expression group. (**D**) The differences of HNF-1B expression percentage between non-pathological transition group and pathological transition group. (**E**) The significant differences of HNF-1B expression level between non-pathological transition group and pathological transition group (Mean±SD).

Representative images of HE staining of the primary tumor and recurrent tumor are illustrated in Fig. 1A. We investigated HNF-1B expression using immunohistochemistry (IHC) in the primary tumor tissue. Representative images of HNF-1B staining of primary tumor in cohort 1 are illustrated in Fig. 1B. The 183 patients were divided into HNF-1B high expression group (2+ and 3+, n = 49) and HNF-1B low expression group (n = 134). In the HNF-1B high expression group, 26.5% (13/49) changed their phenotype on recurrence, but in the HNF-1B low expression group only 1.5% changed the phenotype, 98.5% (132/134) did not change. Chi-square test showed significant statistical difference between the two groups (P < 0.001) (Fig. 1C). Then the 183 patients were divided into a pathological transition group (HCC-ICC, n = 15) and a non-pathological transition group (HCC-HCC, n = 168) based on the transition of recurrence pathological subtype. In sections of the pathological transition group, all patients showed HNF-1B positive staining, with 12 patients in 3+, 1 in 2+ and 2 in 1+. In non-pathological transition group, 67 patients showed HNF-1B positive staining, with 22 patients in 3+, 14 in 2+, 31 in 1+, and 101 in 0 (Fig. 1D). The proportion of HNF-1B high expression cases in HCC-HCC group is 21.4%, much lower than the 86.7% in HCC-ICC group. The level of HNF-1B expression was higher in HCC-ICC group than in HCC-HCC group (P < 0.001, Fig. 1E). These results suggested that HNF-1B may be associated with the transition of recurrence pathological subtype in HCC patients. However, there were 36 HCCs with high HNF1B expression which did not change their phenotype, so the predictive value of HNF1B seems to be poor and there must be other factors associated with the phenotype change, HNF1B may not be the key factor.

### HNF-1B expression levels in different pathologic type of primary tumor

**Figure 2:**
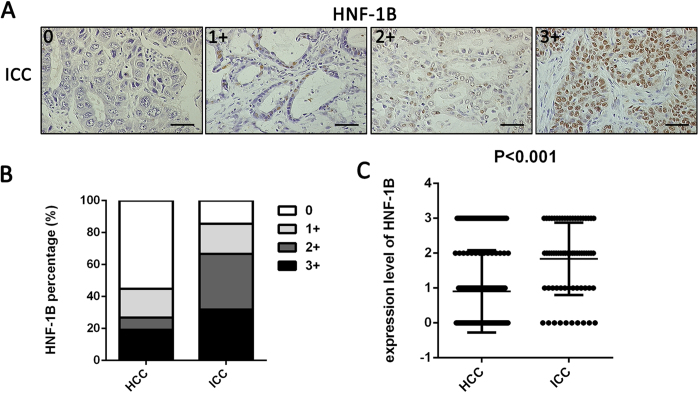
HNF-1B expression is associated with different pathologic types of primary tumor. (**A**) HNF-1B immunohistochemical staining patterns in ICC tissues. Representative sections showing HNF-1B expression in 0, 1+, 2+ and 3 + (sacle bar = 100 μm). (**B**) The differences of HNF-1B expression percentage between HCC patients and ICC patients. (**C**) The significant differences of HNF-1B expression level between HCC patients and ICC patients (Mean±SD).

To investigate the relationship between HNF-1B expression and the pathologic type of the primary tumor, we next investigated HNF-1B expression in primary tumor tissues of 183 HCC patients (cohort1) and 69 ICC patients (cohort2) using IHC. Representative images of HNF-1B staining of ICC are illustrated in Fig. 2A. In HNF-1B staining sections of the 183 HCC patients, 82 patients showed HNF1B positive staining, with 35 patients in 3+, 14 patients in 2+, 33 patients in 1+, and 101 patients in 0. Of the 69 ICC patients, 59 patients showed HNF1B positive staining, with 22 patients in 3+, 24 patients in 2+, 13 patients in 1+, and 10 patients in 0 (Fig. 2B). The level of HNF-1B expression was lower in HCC patients than in ICC patients (*P* < 0.001, Fig. 2C). These results suggested that HNF-1B was associated with different pathologic subtype of primary tumor.

### Association of HNF-1B expression in HCC and ICC with clinicopathological parameters

The baseline characteristics of the 183 HCC patients, 69 ICC patients and the associations of HNF-1B expression with various clinical parameters are shown in Table 2. High expression of HNF-1B was found in 26.8% (49/183) and low expression was found in 73.2% (134/183) of HCC patients. High HNF-1B expression was significantly associated with high TNM stage (*P* = 0.011) and poorly histological grade (*P* = 0.036) in HCC cohort. In the ICC cohort, HNF-1B high expression was found in 66.7% (46/69) and low expression was found in 33.3% (23/69). High HNF-1B expression was significantly associated with lower serum AFP level (*P* = 0.007) in ICC patients.

### The association of HNF-1B expression in HCC with expression of HPCs and biliary markers

**Figure 3:**
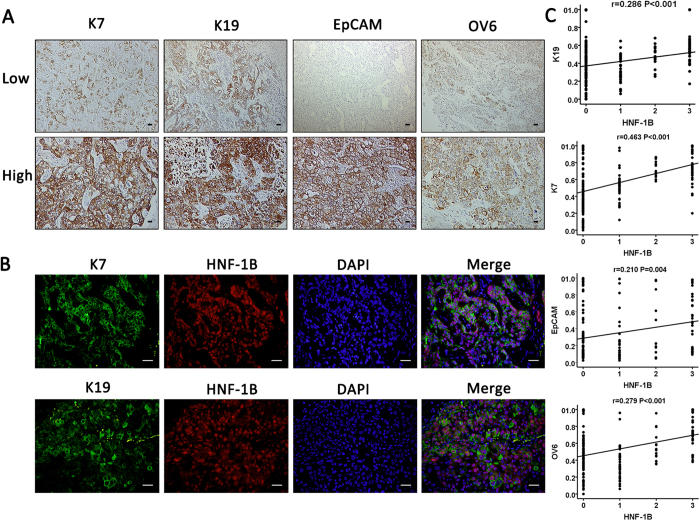
Expression of HNF-1B in HCC is positively correlated with expression of HPCs and biliary markers. (**A**) Representative images of HPCs markers (EpCAM, OV6) and biliary markers (K7, K19) expression (sacle bar = 100 μm). (**B**) Representative images of double staining of HNF-1B and K7, K19 (sacle bar = 100 μm). (**C**) The HNF-1B expression is positively correlated with K7, K19, EpCAM, and OV6 expression. Spearman correlation analysis provides correlation coefficient (r) and P-value.

We next investigated the relationship between HNF-1B and HPC/biliary markers expression in HCC patients. HPCs/biliary markers including cytokeratin 7 (K7), cytokeratin 19 (K19), Epithelial cell adhesion molecule(EpCAM) and OV6 were stained in the HCC tumor tissues. K7, K19, EpCAM and OV6 expression were present in the membrane and cytoplasm of tumor cells. The cutoff value for the definition of subgroups of HPCs markers expression was the median integrated optical density (IOD). The IOD > median was considered as high expression group, and IOD ≤ median was considered as low expression group (Fig. 3A). Double immunostaining showed coexpression of HNF-1B and K7 or K19 (Fig. 3B). Next, we further identify the correlation between HNF1B expression and HPC and biliary markers expression (IOD) in tumor tissues. The Spearman’s correlation analysis found that the expression of HNF-1B in HCC tissue had a positive correlation with expression of K7 (r = 0.463, *P* < 0.001), K19 (r = 0.286, *P* < 0.001), EpCAM (r = 0.210, *P* = 0.004), and OV6 (r = 0.279, *P* < 0.001) (Fig. 3C).

We next analyzed the impact of HPC markers on disease-free survival (DFS) and overall survival (OS) and with clinicopathologic correlation. High K7 expression was significantly associated with cirrhosis (*P* = 0.035), lower serum AFP level (*P* = 0.026) and poorly histological grade (*P* = 0.048). High K19 expression was significantly associated with younger age (*P* = 0.023). High EpCAM expression was significantly associated with younger age (*P* = 0.020), higher serum AFP level (*P* = 0.001) and poorly histological grade (*P* = 0.048). High OV6 expression was significantly associated with cirrhosis (*P* = 0.012)(supplemental table 1). Univariate analysis showed that tumor K7 expression(*P* < 0.001), K19 expression (*P* = 0.013) had a significant impact on OS of HCC patients (supplemental Fig. 1A–B, table 3).

We further examined the HNF-1B expression levels in HCC using a panel of HCC cell lines (SMMC-7721, Huh7, LM3, Hep3B, and HepG2). Real-time PCR study showed that HNF1B expression was higher in Huh7, LM3, Hep3B, HepG2 than in SMMC-7721 (supplemental Fig. 2A). We constructed lentiviral-mediated overexpressed HNF1B in cell line SMMC-7721. When HNF1B expression was significantly overexpressed, the biliary/HPC markers (K7, K19, EpCAM, CD44, Sox9, CD133, CD90) expression were significantly increased (supplemental Fig. 2B).

These results suggested that HNF-1B positive malignant cells may be bipotential cells and retain some progenitor-like characteristics. Also, HNF-1B positive HCC displayed biliary phenotype.

### The association of HNF-1B expression with survival in HCC and ICC patients

**Figure 4:**
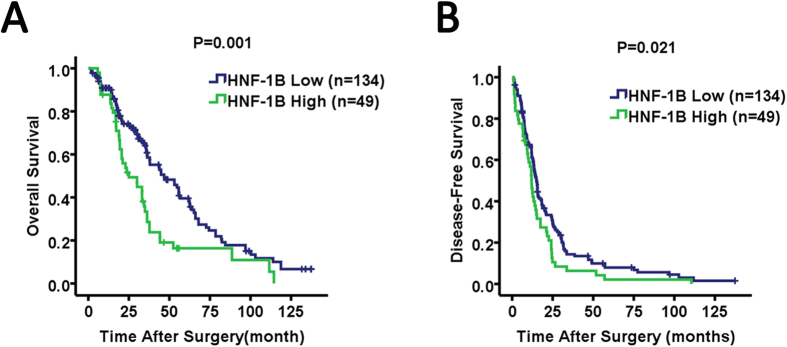
Kaplan-Meier analysis of HNF-1B expression in HCC patients. HNF-1B low expression in HCC tissues was associated with prolonged overall survival (**A**) and disease-free survival (**B**). The P value calculated by the log-rank test is indicated.

In HCC patients, the expression of HNF-1B was significantly associated with OS and DFS (Fig. 4A,B). Patients with high HNF1B expression were likely to have significantly poorer OS and DFS according to Kaplan-Meier survival analysis. The median (95% CI) OS was 46.73 (31.18–62.28) and 25.03 (13.44–36.62) months, respectively, for patients with low HNF-1B expression and high HNF-1B expression in HCC (*P* = 0.001). The median (95% CI) DFS was 15.17 (13.51–16.82) and 11.97 (10.25–13.68) months, respectively, for patients with low HNF-1B expression and high HNF-1B expression in HCC (*P* = 0.021).

In ICC patients, there was no significant differences in OS (*P* = 0.153) and DFS (*P* = 0.891) among the high and low HNF1B expressing group (supplemental table 2 and 3, supplemental Fig. 1C).

### HNF-1B expression in non-tumor tissue of HCC patients and the association with PI-DR and DFS

**Figure 5:**
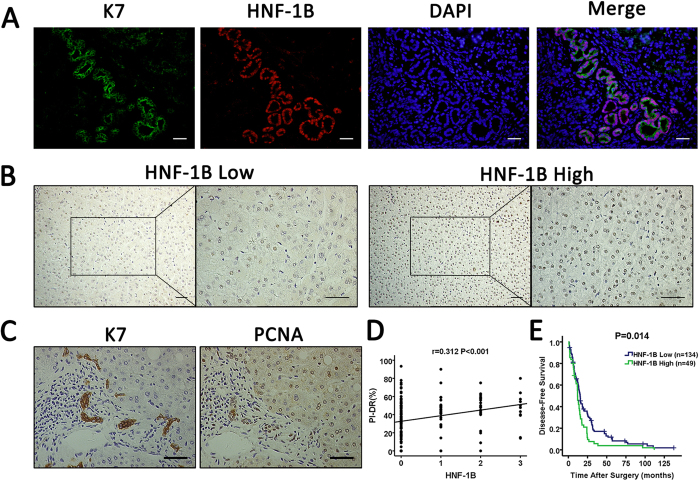
HNF-1B expression in nontumor tissues is associated with PI-DR and DFS. (**A**) Representative images of double staining of HNF-1B and K7 in ductular reaction cells of non-tumor tissue (sacle bar = 100 μm). (**B**) Representative images of HNF-1B expression in hepatocytes of non-tumor tissue. (sacle bar = 100 μm) (**C**) The sequential serial sections stained with K7 and PCNA (sacle bar = 100 μm). (**D**) HNF-1B expression in hepatocytes of non-tumor tissue is significantly correlated with PI-DR. Spearman correlation analysis provides correlation coefficient (r) and P-value. (**E**) HNF-1B low expression in hepatocytes of non-tumor tissues was associated with prolonged disease-free survival. The P value calculated by the log-rank test is indicated.

Next, we analyzed the HNF-1B expression in surrounding non-tumor tissues(>2 cm away from tumor) of the HCC patients. Double immunostaining showed coexpression of HNF-1B and K7 in the ductular reaction cells of non-tumor tissue (Fig. 5A). The representative images of HNF-1B staining of the non-tumor hepatocytes were illustrated in Fig. 5B. In the 183 cases, 67 patients showed HNF-1B positive staining, with 11 patients in 3+, 38 patients in 2+, 18 patients in 1+, and 116 patients in 0. The sequential serial sections stained with K7 and proliferating cell nuclear antigen (PCNA) were shown in Fig. 5C. The proliferation rate in ductular reaction (PI-DR) was calculated as ratio between the number of PCNA immunoreactive nuclei and the total number of K7 positive ductular cells. The Spearman’s correlation analysis found that the expression of HNF-1B in hepatocytes of non-tumor tissue had a positive correlation with PI-DR (r = 0.312, *P* < 0.001) (Fig. 5D). These results suggested that HNF-1B expression is associated with HPC activation in surrounding non-tumor tissues.

The expression of HNF-1B in hepatocytes of non-tumor tissues was also significantly associated with DFS (Fig. 5E). Patients with high HNF-1B expression in non-tumor tissue were likely to be with poor DFS. Kaplan-Meier survival analysis showed that the median (95% CI) DFS time was 15.33 (13.34–17.33) and 11.97 (10.48–13.45) months respectively for patients with low HNF-1B expression and high HNF-1B expression in non-tumor tissue (*P* = 0.014). These results suggested that expression of HNF-1B in surrounding non-tumor tissue was associated with HCC recurrence.

### Univariate and multivariate analyses of prognostic variables in HCC patients

In HCC cohort, the median (95% CI) overall survival time was 36.36(29.89–42.85) months, the minimum time was 1.73 and maximum was 137.4 months. The median (95% CI) disease free survival time was 30.73(25.70–35.77) months, the minimum time was 1.73 and maximum was 137.4 months. Univariate analysis showed that the following factors had a significant impact on OS of HCC patients: higher tumor HNF-1B expression (*P* = 0.001), higher tumor K19 expression (*P* = 0.013), higher tumor K7 expression (*P* < 0.001), larger tumor size (*P* = 0.012), higher TNM stage (*P* = 0.015), and lymphovascular invasion (*P* = 0.008) (Table 3). The following factors had a significant impact on DFS: higher tumor HNF-1B expression (*P* = 0.021), higher TNM stage (*P* = 0.004), larger tumor size (*P* = 0.033), and lymphovascular invasion (*P* = 0.020) (Table 4). In univariate analysis, factors with P < 0.100 were selected for further multivariate Cox proportional hazards analysis.

Multivariate analysis indicated that higher tumor HNF-1B expression (hazard ratio (HR): 1.637, 95% CI: 1.093,2.450, *P* = 0.017), higher tumor K7 expression (HR: 1.799, 95% CI: 1.220–2.654, *P* = 0.003), larger tumor size (HR:1.461, 95% CI: 1.012,2.108, *P* = 0.043), multiple tumor number (HR: 1.708, 95% CI: 1.004–2.904, *P* = 0.048), and lymphovascular invasion (HR: 1.633, 95% CI: 1.101,2.423, *P* = 0.015) were independent prognostic factors for OS (Table 3). Higher tumor HNF-1B expression (HR: 1.443, 95% CI: 1.021–2.039, *P* = 0.038), higher TNM stage (HR: 1.802, 95% CI: 1.057–3.072, *P* = 0.030), and lymphovascular invasion (HR: 1.460, 95% CI: 1.057–2.017, *P* = 0.022) were independent predictors for DFS (Table 4).

### Univariate and multivariable analyses of prognostic variables in ICC patients

In ICC cohort, the median (95% CI) overall survival time was 264.00(221.35–306.65) days, the minimum time was 40.0 and maximum was 411.0 days. The median (95% CI) DFS time was 256.00 (221.94–290.06) days, the minimum time was 26.0 and maximum was 411.0 days. Univariate analysis showed that the following factors had a significant impact on OS of ICC patients: larger tumor size (*P* < 0.001), higher TNM stage (*P* = 0.012), poorly histological grade (*P* = 0.004), and lymphovascular invasion (*P* = 0.035)(supplemental table 2). The following factors had a significant impact on DFS: multiple tumor number (*P* = 0.004), higher TNM stage (*P* = 0.015), and lymphovascular invasion (*P* = 0.049) (supplemental table 3). In univariate analysis, factors with *P* < 0.100 were selected for further multivariate Cox proportional hazards analysis.

Multivariable analysis indicated that larger tumor size (HR: 4.966, 95% CI: 2.348–10.500, *P* < 0.001) and higher TNM stage (HR: 2.660, 95% CI: 1.354–5.228, *P* = 0.005) were independent predictors for OS (supplemental table 2). Multiple tumor number (HR: 3.105, 95% CI: 1.209–7.976, *P* = 0.019) and higher TNM stage (HR: 2.480, 95% CI: 1.001–6.141, *P* = 0.050) were independent predictors for DFS (supplemental table 3).

## Discussion

In this study, we investigated HNF-1B expression in patients with HCC and ICC. The main findings of our study were that HNF-1B expression was associated with the pathologic subtype of primary liver cancer, and HNF-1B expression in HCC tissue may be associated with the change of phenotype on recurrence. HNF-1B expression in HCC patients is positively correlated with the expression of intratumoral HPC/biliary markers. HNF-1B expression in non-tumor tissues positively correlates with proliferative state of ductular reaction (PI-DR) in HCC patients. HNF-1B expression is an independent risk factor for both DFS and OS in HCCs. No correlation between HNF-1B expression and survival was found in ICC patients.

It is long accepted that HCC arises from hepatocyte while ICC is derived from intrahepatic biliary epithelium. However combined hepatocellular-cholangiocarcinoma (CHC), a rare malignant tumor which contains elements of both HCC and ICC, suggesting that HCC and ICC could share the same cellular origin^15^. Hepatic progenitor cells (HPCs) have been identified in the human liver as bipotential cells capable of proliferation and differentiation into both hepatocellular and biliary cell lineages^16^. It is controversial whether CHCs are derived from HPCs. A study also showed that nontumoral PI-DR, which represents HPC activation, was an independent prognostic factor for overall survival and disease free survival in CHC patients after resection^17^. Furthermore, many studies have provided the evidence that liver cancers with high expression of HPC/stem cell markers were of HPC origin and with poor prognosis^12^,^13^,^14^. These results suggested that both HCC and ICC may originate from hepatic stem/progenitor cells. In our study, we found 15 cases who were diagnosed as HCC primarily while their recurrence phenotype changed to ICC. These 15 HCCs may originate from hepatic progenitor cells. Immunohistochemical staining showed positive HNF-1B staining in all of the 15 cases, including 12 cases in 3+, which suggested that HNF-1B may be associated with pathological transition when recurrence. However, the predictive value of HNF1B seems to be poor because there were 36 HCCs with high HNF1B expression which did not change their phenotype on recurrence in our study. There must be other factors associated with the phenotype change, HNF1B may not be the key factor. Comparing HNF-1B expression in HCC with ICC, we found that the level of HNF-1B expression was lower in HCC patients than in ICC patients, which indicates that HNF-1B is associated with the pathologic subtype of primary liver cancer.

HNF-1B has been demonstrated to be associated with the risk of several tumors, including HCC, pancreatic carcinoma, renal cancer, ovarian cancer, endometrial cancer, and prostate cancer^4^,^18^,^19^,^20^,^21^. Recently, studies have provided the evidence that HNF-1B could regulate the expression of genes associated with stem/progenitor cells. In HEK293 cells and ovary clear cell carcinoma, HNF-1B activates the CD24 gene, a cell surface protein that has recently been identified as a marker of the renal progenitor population in the uninduced metanephric mesenchyme^22^. CD24 is often used to identify and enrich cancer stem cells (CSCs) in cancers such as ovarian and pancreatic cancer. It was reported that osteopontin (OPN) is probably a direct target gene of HNF-1B^22^. OPN is overexpressed by liver progenitors in humans and mice. HNF-1B is also reported to regulate the expression of CD44 which is also one of the cell surface markers associated with cancer stem cells in several types of tumor^8^. In pancreas, pancreatic multipotent progenitor cells (MPCs) produce acinar, endocrine and duct cells during organogenesis. HNF-1B has been identified in these pancreatic progenitor cells before differentiation into endocrine or exocrine cells^23^. All of these studies showed that HNF-1B is closely related to stem/progenitor cells in various organs. In our present study, we demonstrated that HNF-1B expression in HCC patients was positively correlated with the expression of intratumoral HPC markers (EpCAM and OV6) and biliary markers (K7, K19). HNF-1B overexpression in HCC cell lines promotes biliary/HPC markers(K7, K19, EpCAM, CD44, Sox9, CD133, CD90) expression. These results suggested that HNF-1B positive malignant cells retain some progenitor-like characteristics, and HNF-1B positive HCC displays biliary phenotype. The immunostaining results of the 15 cases may also suggest that HNF-1B positive malignant cells may be bipotential cells and can give rise to both HCC and ICC. Double immunostaining of HNF-1B and HPC/biliary marker K7 and K19 suggests that HNF1B is expressed in liver progenitor cells. This finding is in consistent with a prior by Rodrigo-Torres *et al.*^24^, their group demonstrated that HNF-1B positive cell population expressed markers of liver progenitor cells. Their research also demonstrated that HNF1B positive biliary duct cells were the origin of LPC, and can differentiate to hepatocytes after certain liver injury.

In liver development, HNF-1B is involved in the hepatobiliary specification of hepatoblasts to cholangiocytes, and it is strongly expressed throughout the embryonic and adult biliary epithelium^24^. In our study, co-staining of HNF-1B and K7 in nontumor tissue demonstrated that HNF-1B is expressed in ductular reaction (DR) cells, which is also consistent with Rodrigo-Torres *et al.*^24^. Ductular reaction is a reactive lesion at the portal tract interface comprising increased bile ductules with an accompanying complex of stromal and inflammatory cells, which has been implicated in the pathogenesis of progressive fibrosis, regeneration and hepato-carcinogenesis in chronic liver disease^25^. In previous studies, we have reported that the proliferative DR (PI-DR) may be involved in the HPC-derived carcinogenesis in the progression of HCC, and could predict recurrence in HCC patients after hepatectomy^25^. Tumor environment is important because of its association with intrahepatic metastasis and recurrence after resection, but is often neglected. In our data, HNF-1B expression in hepatocytes of non-tumor tissue was positively correlated with PI-DR, and was also significantly associated with DFS. These results suggested that HNF-1B could play an important role in HCC recurrence.

In our study, we found no significant difference in DFS and OS among the high and low HNF1B expressing group in ICC patients. This finding was in accord with Mazur *et al.*^26^ that no correlation of HNF1B expression and survival was found in biliary tract cancer. However, there were only 69 patients with ICC in our study, a larger cohort is needed for further study.

In conclusion, we found that in patients with primary liver cancer, the expression of HNF-1B was related to different pathologic subtypes of primary tumor, and HNF-1B expression in HCC tissue may be associated with the change of phenotype on recurrence. In HCC patients, the HNF-1B expression was positively correlated with expression of HPC/biliary markers. HCC with positive HNF-1B expression displayed biliary phenotype. HNF-1B-positive malignant cells could be bipotential cells and give rise to both hepatocytic and cholangiocytic lineages during tumorigenesis. HNF-1B could be a useful marker for prognostic prediction of recurrence in HCC. Further studies are needed to reveal the potential mechanism of HNF-1B during hepatocarcinogenesis.

## Materials and Methods

### Ethics statement

The procedure of human sample acquisition and experiments were approved by the Hospital Research Ethics Committees of the Eastern Hepatobiliary Surgery Hospital of the Second Military Medical University. All methods were carried out in accordance with the approved guidelines.

### Patients and histopathologic analysis

Two independent cohorts of patients were enrolled in this study. Cohort 1 contains 183 HCC patients from 1997 to 2011, cohort 2 contains 69 ICC patients from 2000 to 2007. All patients of the two cohorts were randomly selected from patients with liver cancer who underwent hepatectomy in the Eastern Hepatobiliary Surgery Hospital of the Second Military Medical University. Informed consent was obtained from each patient under a protocol approved by the Hospital Research Ethics Committees. None of the patients had preoperative treatment. The clinicopathological characteristics of patients in the 2 cohorts were summarised in Table 1. For the pathological evaluation, tumor differentiation was assessed according to Edmonson and Steiner grading system. The tumor size was measured macroscopically after the removal of the tumors. Lymphovascular invasion was scored in the tumor and in the surrounding tissue of the tumor. All experimental protocols were conducted in accordance with the Declaration of Helsniki of the World Medical Association.

### Follow-up and detection of recurrence

All patients were followed regularly every 2–3 months after surgery until study closure in July 2013 with serum AFP/CA19-9 and abdominal ultrasonography. Progressive elevation of serum AFP/CA19-9 levels and/or ultrasonographic detection of a new hepatic lesion prompted hospitalization for confirmation of diagnosis and appropriate management, including repeat resection, radiofrequency ablation (RFA), transcatheter arterial chemoembolization (TACE) or supportive therapy. Recurrence was confirmed by contrast-enhanced imaging studies or cholangiography according to standard guidelines for HCC^27^or ICC^28^. Overall survival (OS) was defined as the interval between surgery and death or the last observation taken. The data were censored at the last follow-up period for living patients. Disease free survival (DFS) was defined as the interval between the date of surgery and the date of diagnosis of any type of relapse. If recurrence was not diagnosed, patients were censored on the date of death or the last follow-up.

### Immunohistochemistry and double-fluorescence immunostaining

Formalin-fixed, paraffin-embedded tumor and adjacent nontumor (>2 cm away from tumor) tissues were sectioned at 4-μm intervals. Immunohistochemical staining was carried out on both tumor and nontumor tissue slides. The slides were deparaffinized in xylene and rehydrated through graded alcohol. Endogeneous peroxidase was then inactivated with 3% hydrogen peroxide at room temperature for 20 minutes. Then the slides were soaked in 0.1 mol/L citrate buffer (pH 6.0) and placed in an autoclave at 121 °C for 2 minutes for antigen retrieval. After washing with PBS (pH 7.4), the sections were blocked with 1% BSA diluted in PBS at 37 °C for 30 minutes, and then incubated with primary antibodies at 4 °C overnight. Then the HRP-conjugated goat anti-mouse/rabbit antibody and DAB (DAKO, Glostrup, Denmark) were used. Finally, the sections were counterstained by hematoxylin and mounted. The following antibodies were used: HNF-1B (1:500, Sigma-Aldrich, St. Louis, MO), K7 (1:200, DAKO, Glostrup, Denmark), K19 (1:200, DAKO, Glostrup, Denmark), EpCAM (1:200, DAKO, Glostrup, Denmark), OV6 (1:40, DAKO, Glostrup, Denmark) and PCNA (1:4000, Cell Signaling Technology, MA, USA).

Double-fluorescence immunostaining of the tumor and nontumor tissue was performed with a sequential fluorescent method as described^17^. Immunofluorescence was observed with the Olympus IX-71.

### Evaluation of the immunohistochemical staining

Two independent pathologists without knowledge of patients’ characteristics assessed the immunohistochemical staining. All the staining of slides were observed and photographed with an Olympus microscope (IX-70 OLYMPUS, Japan). We examined a proportion of tumor cells that were positive for HNF-1B in the tumor cells nuclei. We used the criteria to classify and analyze the clinical data; 0 for <5%, 1+ for 5% to 25%, 2+ for 25% to 50%, and 3+ for ≧50%. Cases were considered positive for HNF-1B when nuclear staining was observed in at least 5% (>1+) of the examined tumor cells. Based on the HNF-1B expression, the 183 HCC patients were divided into HNF-1B high group (2+, 3+) and HNF-1B low group (1+, 0). For the staining of HPC markers (K7, K19, EpCAM and OV6), images of positive representative fields were captured under 200x magnification. The density of immunostaining was measured using Image-Pro Plus Version 6.2 software (Media Cybernetics Inc, Bethesda, MD), described previously as an established method^29^. The integrated optical density (IOD) was measured in each image. The cutoff value for the definition of subgroups was the median IOD. The IOD > median was considered as high HPCs markers expression group, and IOD ≤ median was considered as low expression group. The proliferation rate in DR (PI-DR) was evaluated by calculating the proliferating cell nuclear antigen (PCNA) labeling index as described according to Cai *et al.*^17^. The number of reactive ductular cells (RDCs) in reactive ductules was quantificated in sequential serial sections stained with K7^30^. The proliferation index of DR (PI-DR) was calculated as ratio between the number of PCNA immunoreactive nuclei and the total number of RDCs.

### Statistical analysis

Statistical analysis was performed using SPSS software version 12.0 (SPSS, Chicago, IL). Clinicopathologic characteristics in HNF-1B negative and positive cases were compared using Chi-squre test. Differences between categorical variables were assessed by the chi-square test. The degree of association was determined by Spearman correlation. OS and DFS were calculated by Kaplan-Meier method, and differences in survival rate were compared using Log-rank test. Univariate and multivariable analyses were based on the Cox proportional hazards regression model. *P* < 0.05 was considered significant.

## Additional Information

**How to cite this article**: Yu, D.-D. *et al.* Overexpression Of Hepatocyte Nuclear Factor-1beta Predicting Poor Prognosis Is Associated With Biliary Phenotype In Patients With Hepatocellular Carcinoma. *Sci. Rep.* **5**, 13319; doi: 10.1038/srep13319 (2015).

## Supplementary Material

Supplementary Information

## Figures and Tables

**Table 1:** Clinical characteristics of patients with primary liver cancer n (%).

Variable	Cohort1(HCC, n = 183)	Cohort2(ICC, n = 69)	*P* value
Age	49.6 ± 11.0	56.5 ± 11.8	*0.193*
Gender (male/female)	162/21	49/20	0.001*
Tumor number (multiple)	24 (13.1)	12 (17.4)	*0.387*
Maximum tumor diameter	5.2 ± 2.6	6.6 ± 3.2	*0.688*
Lymphovascular invasion	117 (63.9)	27 (39.1)	0.001*
Macrovascular invasion	12 (6.6)	10 (14.5)	0.047*
Cirrhosis	58 (31.7)	21(30.4)	*0.848*
HBsAg (positive)	172(94.0)	37(53.6)	0.001*
Histological grade			**
Well/moderately	133(72.7)	56(81.2)	0.166*
Poorly	50(27.3)	13(18.8)	**
AFP (ng/mL)	399.2(1.7-1000.0)	6.7(1.2-1000.0)	0.001*
CEA (ng/mL)	4.1(0.0-58.2)	4.2(0.0-51.3)	*0.698*
CA19-9 (U/mL)	13.4(0.0-665.0)	40.0(6.0-725.0)	0.001*
ALT (U/L)	53.2(9.8-247.0)	51.2(16.0-238.0)	*0.521*
AST (IU/L)			

AFP, alpha-fetoprotein; CEA, carcinoembryonic antigen; CA19-9, carbohydrate antigen 19–9; ALT, alanine aminotransferase; AST, aspartate aminotransferase; ^*^P *<* *0.05* was considered statistically significant.

**Table 2:** Association of HNF-1B expression with clinicopathologic parameters of HCC and ICC patients.

Variable	HCC		P value	ICC		P value
	Low	High		Low	High	
	(n = 134)	(n = 49)		(n = 23)	(n = 46)	
Age(yr)						
<50	71	25	*0.814*	4	14	*0.245*
≥50	63	24		19	32	
Gender						
Male	118	50	*0.744*	15	34	*0.453*
Female	16	8		8	12	
Tumor size						
<5 cm	118	41	*0.436*	5	17	*0.201*
≥5 cm	16	8		18	29	
Tumor number						
Single	64	25	*0.696*	21	36	*0.178*
Multiple	70	24		2	10	
Cirrhosis						
No	89	36	*0.364*	17	31	*0.579*
Yes	45	13		6	15	
HBsAg						
Negative	9	2	*0.507*	9	23	*0.393*
Positive	125	47		14	23	
TNM stage						
I/II	126	40	0.011*	17	33	*0.849*
III	8	9		6	13	
Serum AFP						
<400	87	29	*0.475*	17	45	0.007*
≥400	47	20		6	1	
Histological grade						
Well/Moderate	103	30	0.036*	18	38	*0.663*
poorly	31	19		5	8	
Lymphovascular invasion						
Negative	46	20	*0.418*	14	28	*1.000*
positive	88	29		9	18	

*P *<* *0.05* was considered statistically significant.

**Table 3:** Univariate and multivariate analysis of OS in HCC patients.

Variable	Number	Univariateanalysis	Multivariatenalysis	
		P value	HR (95% CI)	P value
**Histology:**				
Tumor HNF-1B expression				
Low vs High	134 vs 49	0.001*	1.637(1.093,2.450)	0.017*
Tumor K19 expression				
Low vs High	89 vs 94	0.013*		NS
Tumor K7 expression				
Low vs High	84 vs 99	<0.001*	1.799(1.220,2.654)	0.003*
Tumor EpCAM expression				
Low vs High	95 vs 88	*0.321*		NA
Tumor OV6 expression				
Low vs High	88 vs 95	*0.509*		NA
Non-tumor HNF-1B expression				
Low vs High	134 vs 49	0.026*		NS
**Clinical characteristics**				
Age(yr)				
<50 vs ≥50	96 vs 87	*0.129*		NA
Gender				
Male vs Female	162 vs 21	*0.924*		NA
Tumor size				
<5 cm vs ≥5 cm	89 vs 94	0.012*	1.461(1.012,2.108)	0.043*
Tumor number				
Single vs Multiple	159 vs 24	*0.071*	1.708(1.004,2.904)	0.048*
Cirrhosis				
No vs Yes	125 vs 58	*0.308*		NA
TNM stage				
I/II vs III	166 vs 17	0.015*		NS
HbsAg				
Negative vs Positive	11 vs 172	*0.606*		NA
Serum AFP				
<400 vs ≥400	116 vs 67	*0.428*		NA
Histological grade				
Well/moderate vs poorly	133 vs 50	*0.592*		NA
Lymphovascular invasion				
Negative vs Positive	66 vs 117	0.008*	1.633(1.101,2.423)	0.015*

NA, not adopted; NS, not significant; HR, hazard ratio; Factors with p < 0.1 in univariate analysis were adopted for further multivariate analysis. *P *<* *0.05* was considered statistically significant.

**Table 4:** Univariate and multivariate analysis of DFS in HCC patients.

Variable	Number	Univariateanalysis	Multivariatenalysis	
		P value	HR (95% CI)	P value
**Histology:**				
Tumor HNF-1B expression				
Low vs High	134 vs 49	0.021*	1.443(1.021,2.039)	0.038*
Tumor K19 expression				
Low vs High	89 vs 94	*0.128*		NA
Tumor K7 expression				
Low vs High	84 vs 99	*0.051*		NS
Tumor EpCAM expression				
Low vs High	95 vs 88	*0.684*		NA
Tumor OV6 expression				
Low vs High	88 vs 95	*0.793*		NA
Non-tumor HNF-1B expression				
Low vs High	134 vs 49	0.014*		NS
**Clinical characteristics**				
Age(yr)				
<50 vs ≥50	96 vs 87	*0.738*		NA
Gender				
Male vs Female	162 vs 21	*0.695*		NA
Tumor size				
<5 cm vs ≥5 cm	89 vs 94	0.033*		NS
Tumor number				
Single vs Multiple	159 vs 24	*0.433*		NA
Cirrhosis				
No vs Yes	125 vs 58	*0.110*		NA
TNM stage				
I/II vs III	166 vs 17	0.004*	1.802(1.057,3.072)	0.030*
HbsAg				
Negative vs Positive	11 vs 172	*0.105*		NA
Serum AFP				
<400 vs ≥400	116 vs 67	*0.246*		NA
Histological grade				
Well/moderate vs poorly	133 vs 50	*0.514*		NA
Lymphovascular invasion				
Negative vs Positive	66 vs 117	0.020*	1.460(1.057,2.017)	0.022*

NA, not adopted; NS, not significant; HR, hazard ratio; Factors with p < 0.1 in univariate analysis were adopted for further multivariate analysis. *P *<* *0.05* was considered statistically significant.
