# Supplementary material for: Overexpression Of Hepatocyte Nuclear Factor-1beta Predicting Poor Prognosis Is Associated With Biliary Phenotype In Patients With Hepatocellular Carcinoma
Source: Sci Rep. 2015 Aug 27;5:13319. doi: 10.1038/srep13319 (PMC4550878; doi:10.1038/srep13319)

Overexpression Of Hepatocyte Nuclear Factor-1beta Predicting Poor Prognosis Is Associated With Biliary Phenotype In Patients With Hepatocellular Carcinoma

Authors: Dan-Dan Yu[1#], Ying-Ying Jing[1#], Shi-Wei Guo[1#], Fei Ye[1], Wen Lu[1], Quan Li[1], Yu-Long Dong[1], Lu Gao[1], Yu-Ting Yang[1], Yang Yang[1], Meng-Chao Wu[2*], Li-Xin Wei[1*]

Author's affiliations:

[1]Tumor Immunology and Gene Therapy Center, Eastern Hepatobiliary Surgery Hospital, The Second Military Medical University, Shanghai, China,

[2]Department of Comprehensive Treatment, Eastern Hepatobiliary Surgery Hospital, The Second Military Medical University, Shanghai, China,

***Corresponding author**: Li-Xin Wei, Tumor Immunology and Gene Therapy Center, Eastern Hepatobiliary Surgery Hospital, The Second Military Medical University, 225 Changhai Road, Shanghai, 200438 China, Tel.: +86 21 81875331; Fax: +86 21 65566349; E-mail: weilixin_smmu@163.com.

And

Meng-Chao Wu, Department of Comprehensive Treatment, Eastern Hepatobiliary Surgery Hospital, The Second Military Medical University, NO. 225 Changhai Road, Shanghai, 200438 China, E-mail: wuyuz@yahoo.com.cn

[#]These authors contributed equally to this work.

Supplemental Tables

Supplemental table 1. HPC/biliary markers expression and clinicopathologic features in tumor tissue of 183 HCCs

Clinicopathologic features	K7		P value	K19		P value	EpCAM		P value	OV6		P value
	Low	High		Low	High		Low	High		Low	High	
Frequency(%)	84 (45.9)	99 (54.1)		89 (48.6)	94 (51.4)		95 (51.9)	88 (48.1)		88 (48.1)	95 (51.9)	
Age(yr)												
＜50	46	50	0.566	39	57	0.023*	42	54	0.020*	42	54	0.217
≥50	38	49		50	37		53	34		46	41	
Gender												
Male	76	86	0.445	76	86	0.196	84	78	0.964	74	88	0.070
Female	8	13		13	8		11	10		14	7	
Tumor size												
＜5cm	42	47	0.733	48	41	0.163	50	39	0.261	49	40	0.066
≥5cm	42	52		41	53		45	49		39	55	
Tumor number												
Single	74	85	0.655	79	80	0.464	87	72	0.051	77	82	0.813
Multiple	10	14		10	14		8	16		11	13	
Cirrhosis												
No	64	61	0.035*	64	61	0.308	68	57	0.323	68	57	0.012*
Yes	20	38		25	33		27	31		20	38	
HBsAg												
Negative	5	6	0.976	8	3	0.099	3	8	0.092	4	7	0.422
Positive	79	93		81	91		92	80		84	88	
TNM stage												
Ⅰ/Ⅱ	80	86	0.052	82	84	0.518	89	77	0.150	80	86	0.929
Ⅲ	4	13		7	10		6	11		8	9	
Serum AFP												
＜400	46	70	0.026*	53	63	0.294	71	45	0.001*	52	64	0.246
≥400	38	29		36	31		24	43		36	31	
Histological grade												
Well /Moderate	67	66	0.048*	69	64	0.152	75	58	0.048*	67	66	0.312
poorly	17	33		20	30		20	30		21	29	
Lymphovascular invasion												
Negative	34	32	0.252	37	29	0.131	37	29	0.399	35	31	0.315
Positive	50	67		52	65		58	59		53	64	

*$P<0.05$ was considered statistically significant.

Supplemental table 2. Univariate and multivariate analysis of OS in ICC patients

Variable	Number	Univariate analysis	Multivariate analysis	
		P value	HR (95% CI)	P value
**Histology:**				
Tumor HNF-1B expression				
Low vs High	23 vs 46	*0.153*		NA
Non-tumor HNF-1B expression				
Low vs High	43 vs 26	*0.298*		NA
**Clinical characteristics**				
Age(yr)				
＜50 vs ≥50	18 vs 51	*0.252*		NA
Gender				
Male vs Female	49 vs 20	*0.227*		NA
Tumor size				
＜5cm vs ≥5cm	22 vs 47	*<0.001**	4.966(2.348,10.500)	*<0.001**
Tumor number				
Single vs Multiple	57 vs 12	*0.169*		NA
Cirrhosis				
No vs Yes	48 vs 21	*0.868*		NA
TNM stage				
Ⅰ/Ⅱ vsⅢ	50 vs 19	*0.012**	2.660(1.354,5.228)	*0.005**
HBsAg				
Negative vs Positive	32 vs 37	*0.681*		NA
Serum AFP				
＜400 vs ≥400	62 vs 7	*0.527*		NA
Histological grade				
Well / moderate vs poorly	56 vs 13	*0.004**		NS
Lymphovascular invasion				
Negative vs Positive	42 vs 27	*0.035**		NS

NA, not adopted; NS, not significant; HR, hazard ratio; Factors with p<0.1 in univariate analysis were adopted for further multivariate analysis. *P<0.05 was considered statistically significant.

Supplemental table 3. Univariate and multivariate analysis of DFS in ICC patients

Variable	Number	Univariate analysis	Multivariate analysis	
		P value	HR (95% CI)	P value
**Histology:Tumor**				
Tumor HNF-1B expression				
Low vs High	23 vs 46	*0.891*		NA
Non-tumor HNF-1B expression				
Low vs High	43 vs 26	*0.595*		NA
**Clinical characteristics**				

Age(yr)				
＜50 vs ≥50	18 vs 51	*0.138*		NA
Gender				
Male vs Female	49 vs 20	*0.588*		NA
Tumor size				
＜5cm vs ≥5cm	22 vs 47	*0.643*		NA
Tumor number				
Single vs Multiple	57 vs 12	*0.004**	3.105(1.209,7.976)	*0.019**
Cirrhosis				
No vs Yes	48 vs 21	*0.355*		NA
TNM stage				
Ⅰ/Ⅱ vsIII	50 vs 19	*0.015**	2.480(1.001,6.141)	*0.050**
HBsAg				
Negative vs Positive	32 vs 37	*0.541*		NA
Serum AFP				
＜400 vs ≥400	62 vs 7	*0.461*		NA
Histological grade				
Well / moderate vs poorly	56 vs 13	*0.057*		NS
Lymphovascular invasion				
Negative vs Positive	42 vs 27	*0.049**		NS

NA, not adopted; NS, not significant; HR, hazard ratio; Factors with $p<0.1$ in univariate analysis were adopted for further multivariate analysis. *$P<0.05$ was considered statistically significant.

Supplemental Materials and methods

Cell culture and tumor cell infection

The human hepatocellular carcinoma cell lines HepG2, Huh7, Hep3B, LM3 and SMMC-7721 were obtained from the Cell Bank of Type Culture Collection of Chinese Academy of Sciences, Shanghai Institute of Cell Biology, Chinese Academy of Sciences. HepG2, Huh7, Hep3B, LM3 and SMMC-7721 cells were cultured in Dulbecco’s modified Eagle’s medium (high glucose) (Gibco, Grand Island, NY, USA) containing 10% fetal bovine serum(FBS) and 100 U/ml of penicillin/streptomycin (Gibco). All cells were incubated in a humidified incubator at 37 ℃ with 5% CO_2 and 95% air. Cells ($1–3\times10^6$) grown to 50–60% confluence in 10cm Petri dishes were transfected with HNF-1B lentiviral expression vector or its corresponding mock sequences.

RNA extraction and real-time quantitative PCR

Total RNA extraction, complementary DNA (cDNA) synthesis, and qPCR were performed as described previously[1]. The primer sequences used in the qPCR are shown in Supplemental table 4.

Supplemental table 4. Sequences of RT-PCR oligonucleotide primers

		Sequence (5'→3')
HNF-1B	F	GTGGACCGGATGCTCAGTG
	R	GGGTCTTCATAGGGGTGCC
K7	F	TCCGCGAGGTCACCATTAAC
	R	GCTCTGTCAACTCCGTCTCAT
K19	F	ACCAAGTTTGAGACGGAACAG
	R	CCCTCAGCGTACTGATTTCCT
EpCAM	F	AATCGTCAATGCCAGTGTACTT
	R	TCTCATCGCAGTCAGGATCATAA
Sox9	F	AGCGAACGCACATCAAGAC
	R	CTGTAGGCGATCTGTTGGGG
CD133	F	AGTCGGAAACTGGCAGATAGC
	R	GGTAGTGTTGTACTGGGCCAAT
CD44	F	CTGCCGCTTTGCAGGTGTA
	R	CATTGTGGGCAAGGTGCTATT
CD90	F	ATCGCTCTCCTGCTAACAGTC
	R	CTCGTACTGGATGGGTGAACT
DAPDH	F	TGCCAAATATGATGACATCAAGAA
	R	GGAGTGGGTGTCGCTGTTG

Supplemental References

1 Sun, T. *et al.* Expression and functional significance of Twist1 in hepatocellular carcinoma: its role in vasculogenic mimicry. *Hepatology* **51**, 545-556, doi:10.1002/hep.23311 (2010).

Supplemental Figure legends

Supplemental Figure 1. Kaplan-Meier analysis of K7 and K19 expression in HCC patients and HNF-1B expression in ICC patients.

K7 and K19 low expression in HCC tissues was associated with prolonged overall

survival (A) and (B). No significant differences in DFS among the high and low K7/K19 expressing group was found in HCC patients. (C) No significant differences in OS among the high and low HNF1B expressing group was found in ICC patients. The P value calculated by the log-rank test is indicated.

Supplemental Figure 2. Overexpression of HNF-1B in HCC cell lines increase biliary/HPC markers expression

(A)HNF-1B mRNA levels were different in HCC cell lines Huh7, LM3, Hep3B, HepG2 and SMMC-7721. (B) SMMC-7721 cells were transfected with lentiviral vectors or its corresponding mock sequences to overexpress HNF-1B. RT-PCR was used to detect changes in the expression of biliary/HPC markers. Results represent means of triplicate experiments ± standard errors (*$P<0.05$).

Supplemental Figures

Supplemental Figure 1

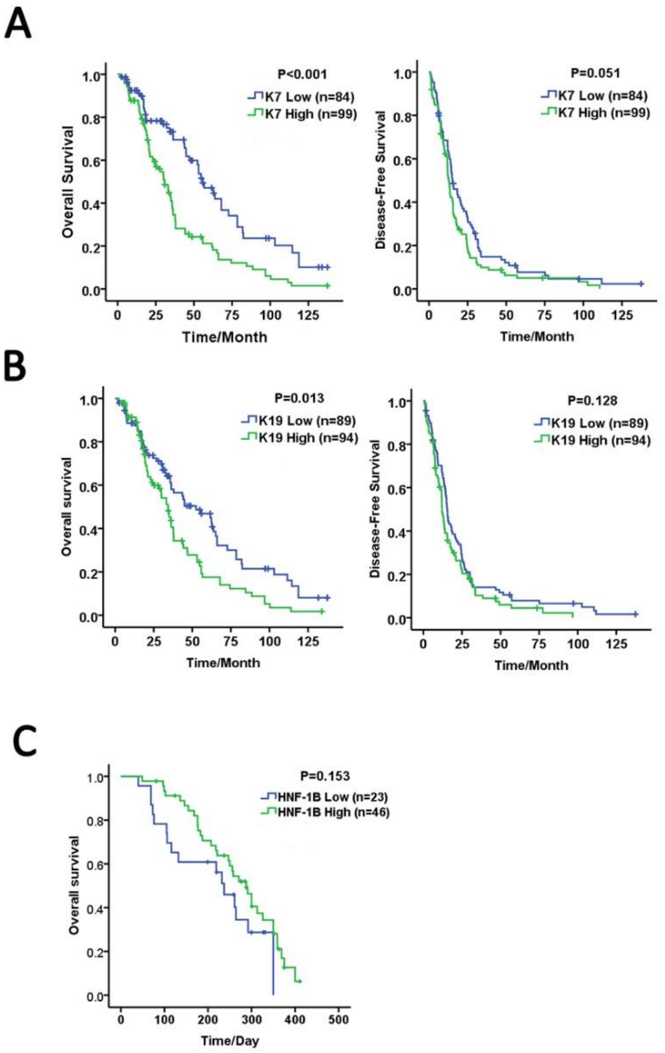
A
P<0.001
K7 Low (n=84)
K7 High (n=99)
Overall Survival
Time/Month
P=0.051
K7 Low (n=84)
K7 High (n=99)
Disease-Free Survival
Time/Month
B
P=0.013
K19 Low (n=89)
K19 High (n=94)
Overall survival
Time/Month
P=0.128
K19 Low (n=89)
K19 High (n=94)
Disease-Free Survival
Time/Month
C
P=0.153
HNF-1B Low (n=23)
HNF-1B High (n=46)
Overall survival
Time/Day

Supplemental Figure 2

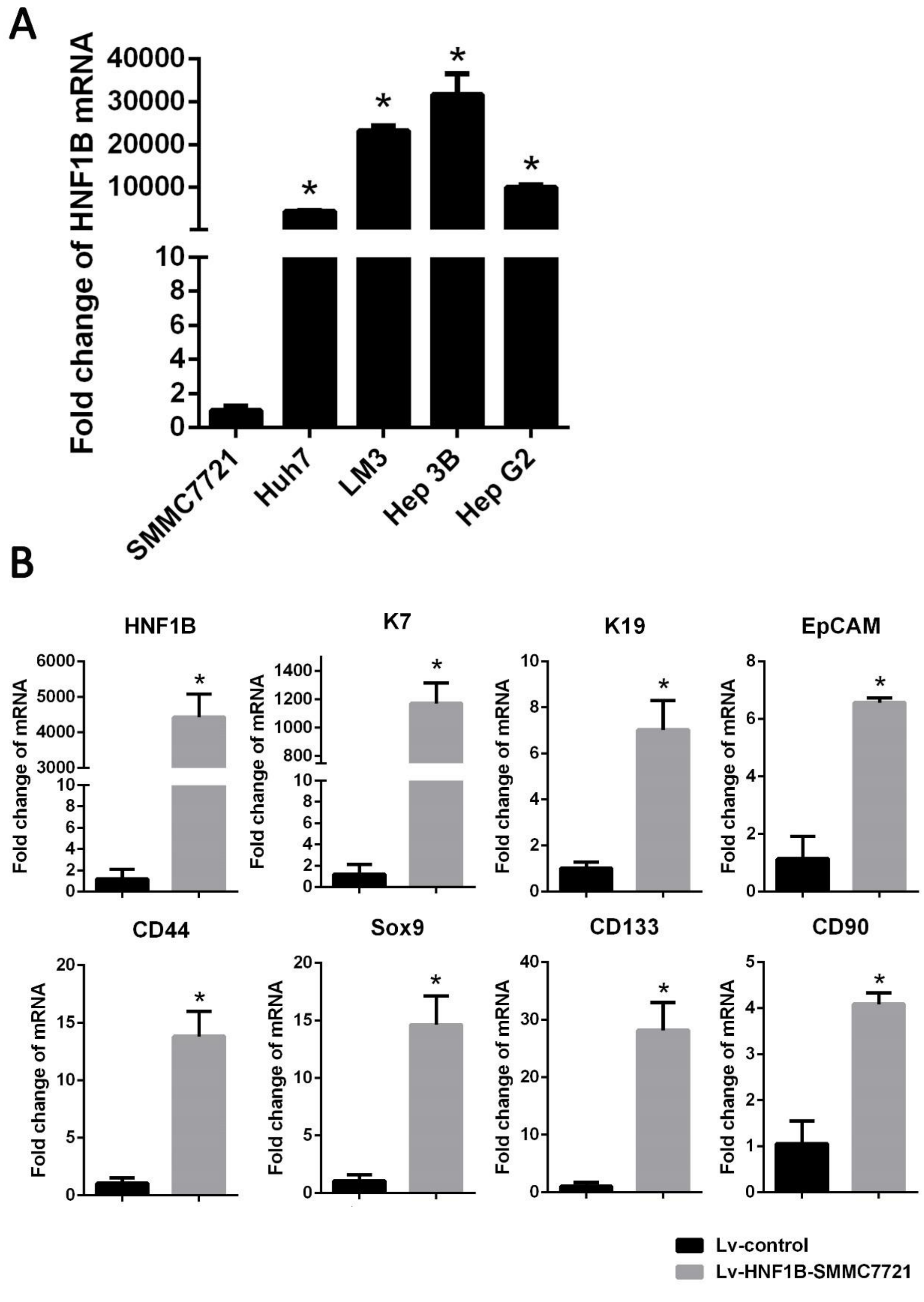

Supplement: Supplementary Information [file srep13319-s1.pdf]
